# Upper Limb Meromelia with Oligodactyly and Brachymesophalangy of the Foot: An Unusual Association

**DOI:** 10.1155/2019/3419383

**Published:** 2019-06-24

**Authors:** Meltem Özdemir, Rasime Pelin Kavak, Önder Eraslan

**Affiliations:** University of Health Sciences, Dışkapı Yıldırım Beyazıt Training and Research Hospital, Department of Radiology, Ankara, Turkey

## Abstract

Meromelia is a rare skeletal abnormality characterized by the partial absence of at least one limb. Several mechanisms have been postulated to explain the etiopathogenesis of the disorder. Most of the cases of meromelia are reported to be sporadic. It can occur either in isolation or with other congenital malformations. VACTERL association, gastroschisis, atrial septal defect, proximal femoral focal deficiency, and fibular hemimelia are the congenital abnormalities reported to be in association with meromelia. However, no other congenital abnormalities in association with meromelia have been recorded to date. We herein present an unusual case of bilateral upper limb meromelia accompanied by unilateral oligodactyly and brachymesophalangy of the foot.

## 1. Introduction

Amelia refers to the complete absence of at least one limb, and meromelia is characterized by the partial absence of at least one limb. Meromelia is also termed as “terminal tarsverse hemimelia”. Actually, amelia and meromelia are two forms of the same disorder which are in continuation with each other in terms of severity [1, 2]. Therefore, the term “amelia-meromelia sequence” is also used to define the limb reduction of this type [3]. It is a rare abnormality with a prevalence of 1.41 per 100,000 births. Several mechanisms by which the limb deficiencies can occur have been described in order to explain the etiology of the disorder. The development of upper limbs begins on the 26th day following fertilization. An insult during this period or genetic errors in the coordination of limb development can result in the failure of the limb formation in a very early stage of embryonic life. Further, intrauterine amputation as a cause of amniotic bands or deterioration of the arterial supply to the limb may also cause limb reduction [4]. Most of the cases are reported to be sporadic and nontransmissible. Meromelia can occur in either isolation or with other congenital malformations [3]. In literature, there are very few reports of meromelia in association with other congenital anomalies [3, 5, 6]. We herein present an unusual case of meromelia accompanied by congenital deformity of the foot.

## 2. Case Report

A 20-year-old man with both upper limb disabilities admitted to our hospital for mandatory health screening before military service. He had no health complaints other than the skeletal disorder involving his upper limbs. In detailed questioning, he stated that he had four fingers in his left foot. He is the last of six children born to nonconsanguineous healthy parents. All of his brothers and sisters are completely healthy. There is no family history of any kind of congenital skeletal abnormalities in the extended family. His mother was at the age of 32 when she gave birth to our patient. There is no history of any drug, smoke, alcohol, or radiation exposure during pregnancy. Our patient was born uneventfully at full-term through normal vaginal delivery. No other significant health problem is present in the history of his childhood.

**Figure 1:**
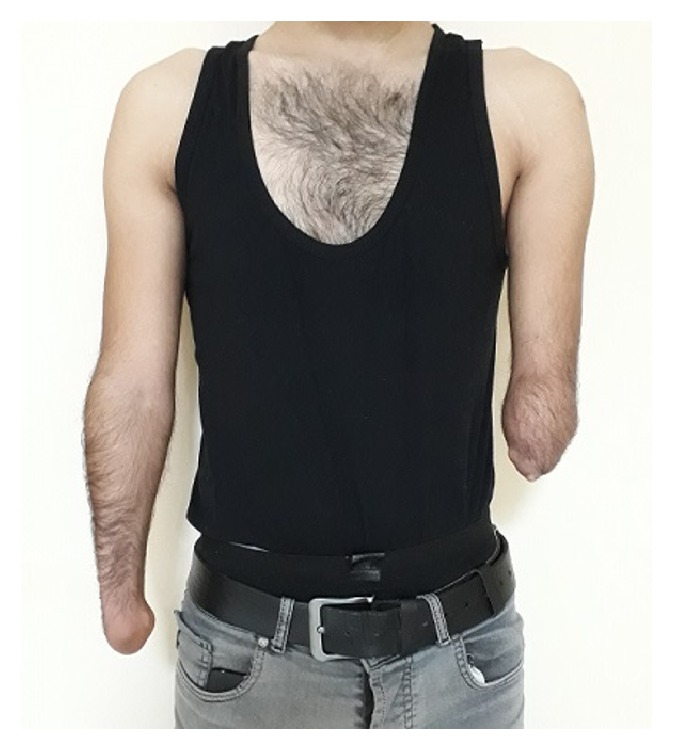
Clinical photograph of the patient with bilateral upper limb meromelia.

**Figure 2:**
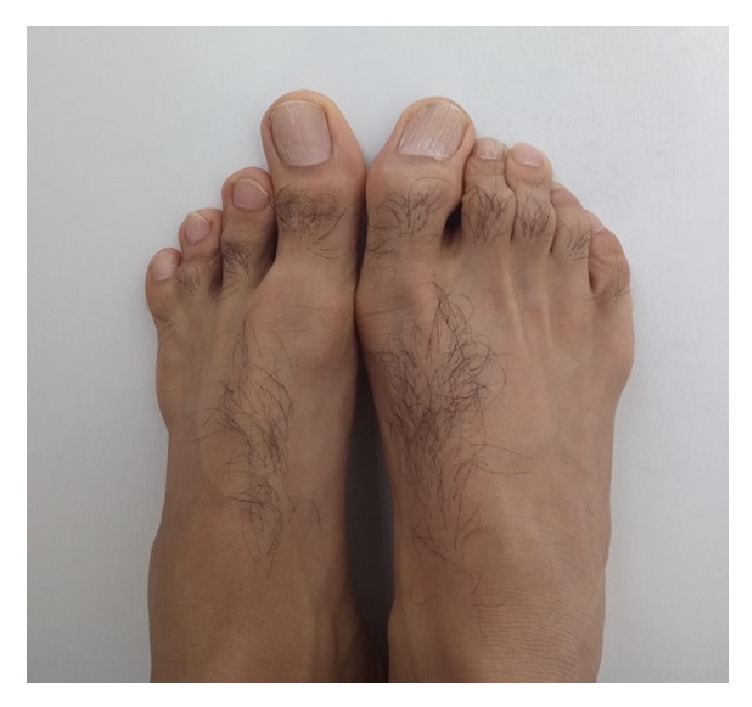
Clinical photograph of the patient with oligodactyly of the left foot.

**Figure 3:**
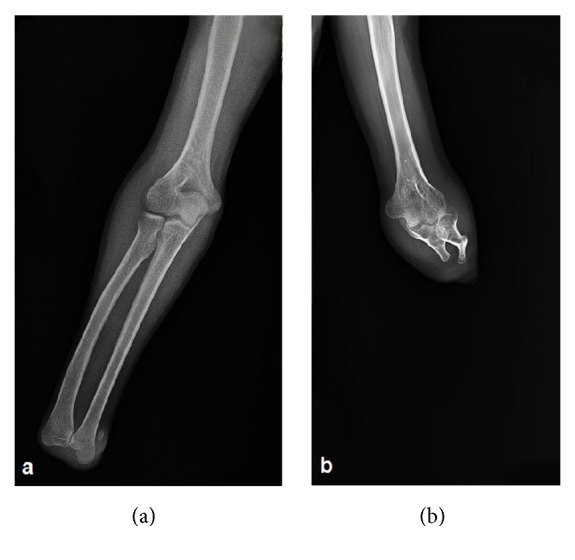
Anteroposterior radiographs of the right (a) and the left (b) arms demonstrating the absence of the right hand with well-developed radius and ulna, and the absence of the left forearm distal to the elbow where there are small radial and ulnar segments participating in the elbow joint. There is a rudimentary bone of about 1 cm in the medial neighborhood of the medial aspect of the right distal ulna.

**Figure 4:**
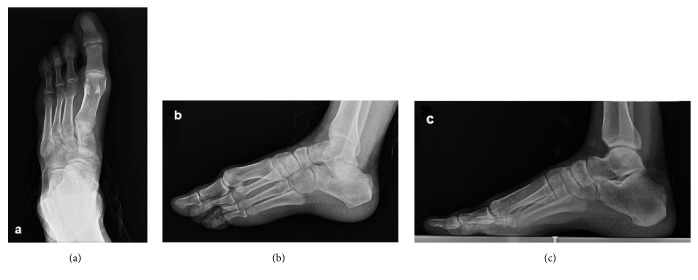
Anteroposterior (a), lateral oblique (b), and mediolateral (c) radiographs of the left foot demonstrate the absence of the fifth finger and the lateral cuneiform. The second to fourth fingers are short. The number, size, and joint relations of the other bones forming the foot are normal.

**Figure 5:**
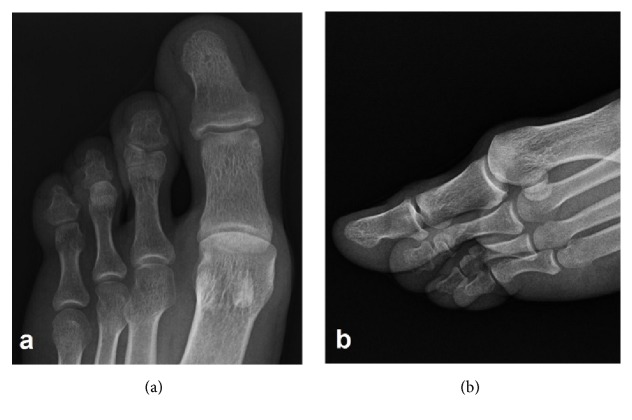
Magnified anteroposterior (a) and lateral oblique (b) radiographs of the left foot show that the middle phalanges of the second and third fingers are shorter than the distal phalanges, and the middle phalanx of the fourth finger is absent.

On physical examination, all skeletal elements beyond his left elbow and right wrist, and the fifth finger of his left foot were found to be absent (Figure 1). He had oligodactyly of the left foot (Figure 2). Anteroposterior radiograph of the right arm depicted the absence of the hand with well-developed radius and ulna. There was a rudimentary bone of about 1 cm in the medial neighborhood of the distal ulna (Figure 3(a)). Anteroposterior radiograph of the left arm demonstrated that the proximal forearm segment participating in the elbow joint structure was small but present, while the radius and ulna distal to this point were absent (Figure 3(b)). Anteroposterior, lateral oblique, and mediolateral radiographs of the left foot revealed absence of the fifth finger and the lateral cuneiform. The second to fourth fingers were short. The number, size, and joint relations of the other bones forming the foot were normal (Figure 4). Magnified anteroposterior and lateral oblique radiographs of the left foot showed that the middle phalanges of the second and third fingers were shorter than the distal phalanges, and the middle phalanx of the fourth finger was absent (Figure 5).

The patient was then referred to the Department of Orthopedic Surgery to identify the best prosthetic fitting option and to provide a convenient training regimen.

## 3. Discussion

In a recent epidemiologic study which was carried out by evaluating the large data set collected by 20 surveillance programs on congenital anomalies, other congenital anomalies were shown to accompany 66.9% of the cases with amelia. The most frequent abnormalities associated with amelia were other types of musculoskeletal anomalies, intestinal, renal, genital defects, cardiac septal defects, oral clefts, and anencephaly. According to the results of this comprehensive study, congenital feet deformities were present in 13.8% of all cases with amelia [4]. However, reports of meromelia cases accompanied by other congenital anomalies are extremely rare in the current literature. Anomalies of vertebral, anal, cardiac, tracheo-esophageal, radial, and limb (VACTERL) association [5], gastroschisis [6], and atrial septal defect [3] are the congenital abnormalities reported to be in association with meromelia. And, musculoskeletal anomalies reported to accompany meromelia are proximal femoral focal deficiency and fibular hemimelia [5]. No congenital feet deformity in association with meromelia has been recorded to date.

Congenital foot oligodactyly is referred to the absence of one or more toes. Lateral rays are reported to be affected more often than the hallux. It can be associated with fibular hemimelia, tarsal coalition, and other foot anomalies [7]. Brachymesophalangy is a rare skeletal abnormality which is characterized by the absence or hypoplasia of the middle phalanx. The term is used when the middle phalanges have the same length or are shorter than the distal phalanges. It is one of the five major types of brachydactyly according to Bell's classification [8]. There are six subtypes of brachymesophalangy all of which are autosomal dominant inheritable disorders with well-described phenotypical characteristics [9]. Our patient falls outside this classification as all of his extended family members are healthy and he does not present phenotypical characteristics of any subtype of brachymesophalangy. The disorder can rarely be found in isolation, or other limb abnormalities such as synbrachydactyly [10, 11].

Although this is the first case report of meromelia associated with foot abnormality, we think it may not be a rare association. Considering the fact that all of the previously reported patients with meromelia were in the neonatal stage of life and taking the difficulty of diagnosing a minor skeletal abnormality such as brachymesophalangy in a tiny individual in consideration, the association between the two anomalies may have been easily overlooked in the previously reported cases. A survey of minor skeletal abnormalities after reaching a certain level of maturation of hand and foot bones would be appropriate in newborn cases with amelia and meromelia.

Brachymesophalangy is a minor skeletal abnormality which usually does not cause functional disturbances. Likewise, with the full usage of the remaining digits, foot oligodactyly does not give rise to difficulty in daily activities. However, causing not only a significant physical disability but also a serious cosmetic discomfort, bilateral upper limb meromelia requires a comprehensive treatment approach. Supplementation of a prothesis or orthosis is indicated in the rehabilitation of children with limb deficiencies. A prothesis replaces parts of the extremities, whereas an orthosis stabilizes the existing extremity [12]. Our patient was offered prosthetic instrumentation for several times during his childhood. However, because of the financial difficulties and his family's inability to show sufficient care, he had never undertaken a suitable training or been provided a prothesis. He was experiencing serious trouble in performing daily activities. He did not even have the ability to get dressed and undressed on his own. The other issues which have to be addressed in the management of the patients with limb reduction and are at least as important as prosthetic instrumentation are the need for training and the psychological situation of the child. Therefore, the management of these cases should be carried out in interdisciplinary and specialized clinical settings. A team approach of which the members are the physician, orthopedist, prosthetist, physical therapist, occupational therapist, psychologist, and the mother of the child have shown to be the most successful approach in raising the life quality of the handicapped child [13–15].
